# Effects of Adhesive Bond-Slip Behavior on the Capacity of Innovative FRP Retrofits for Fatigue and Fracture Repair of Hydraulic Steel Structures

**DOI:** 10.3390/ma12091495

**Published:** 2019-05-08

**Authors:** Christine M. Lozano, Guillermo A. Riveros

**Affiliations:** United States Army Corps of Engineers Engineer Research and Development Center, Vicksburg, MS 39180-6199, USA

**Keywords:** carbon fibers, fatigue and fracture repairs, hydraulic steel structures, extended finite element, bond-slip, traction-separation, cohesive damage

## Abstract

Over eighty percent of the navigation steel structures (NSS) in the United States have highly deteriorated design boundary conditions, resulting in overloads that cause fatigue cracking. The NSSs’ highly corrosive environment and deterioration of the protective system accelerate the fatigue cracking and cause standard crack repair methods to become ineffective. Numerous studies have assessed and demonstrated the use of carbon fiber reinforced polymers (CFRP) to rehabilitate aging and deteriorated reinforced concrete infrastructure in the aerospace industry. Due to the increase of fatigue and fracture failures of NSS and the shortage of research on CFRP retrofits for submerged steel structures, it is imperative to conduct research on the effects of CFRP repairs on NSS, specifically on the adhesive’s chemical bonding to the steel substrate. This was accomplished by developing a new analytical algorithm for CFRP bond-slip behavior, which is based on Volkersen’s contact shear single lap joint (SLJ) connection. The algorithm was validated by experimental results of fatigue center-cracked large steel plates repaired with CFRP patches. The state of stresses at the crack tip are largely influenced by a combination of the crack tip plasticity radius and overall bond surface area.

## 1. Introduction

Over eighty percent of the navigation steel structures (NSS) within the United States are near to or exceeding their design life [1,2,3]. Due to deterioration over time and cyclic operation in submerged, highly corrosive environments, NSSs are experiencing fatigue and cracking. For the majority of their life, NSSs are mostly submerged in murky water, therefore the fatigue and fractures, most occurring in the lower/submerged portion of the NSS, go unobserved until they lead to a failure or a scheduled inspection or maintenance. For example, in 2014, Pickwick Lock and Dam operation’s staff began to observe longer pool rise times and noise, prompting an inspection. The inspection required a full dewater of the valve. During the inspection, a full fracture of the welded connection of the strut arm’s wide flange was discovered and repaired. The implemented repair was a worse fatigue life category detail that required replacement only two years later. An unscheduled operation stoppage costs the U.S. Army Corps of Engineers 1–3 million dollars per day [4,5,6]. As time passes with gates kept in operation, and no yearly maintenance, the probability of a fatigue and fracture failure and unscheduled stoppage increases linearly [4,7].

The majority of fatigue and fracture repair methods were developed for Mode I cracking in the bridge industry [8,9,10,11]. Due to the mixed mode stress states and submerged environment, NSSs require a different repair method. NSSs require a repair or retrofit that can operate in a submerged environment, will not require further welding or holes (potentials for new crack growth), and is flexible enough to be applied to the lock gate’s many different geometrical sections. 

Over the past five years, several NSSs’ have had fatigue and fractures repaired with a new repair method, carbon fiber reinforced polymer (CFRP) patches [8,9]. This research is part of a five-year project to extend the useful fatigue life of NSS. The conclusion of this research is to develop guidelines to enhance the fatigue life of NSS, which will be incorporated in USACE design memorandums. CFRP has a high strength capacity, low density, and is versatile, making it ideal for fatigue and fracture repairs [10]. To implement the repairs, a lock chamber must be dewatered and the metal section grinded to the base metal, ending with the application of the CFRP composite. The patches are applied to the cracked section using a high-strength adhesive. The adhesive itself is weaker than the steel or CFRP. Therefore, the adhesive’s bond strength and behavior are foundational to the overall performance of the CFRP retrofit. This article will describe the bond behavior between steel and adhesive. 

This article develops an analytical equation, utilizing Volkersen’s contact shear single lap joint (SLJ) connection [12], to determine the fatigue life increase due to the strength contribution of CFRP in the x and y directions. During crack growth, contact shear stresses are developed in the x and y directions, due to the x and y stresses generated by the crack. 

### 1.1. Fatigue and Fracture

**Figure 1:**
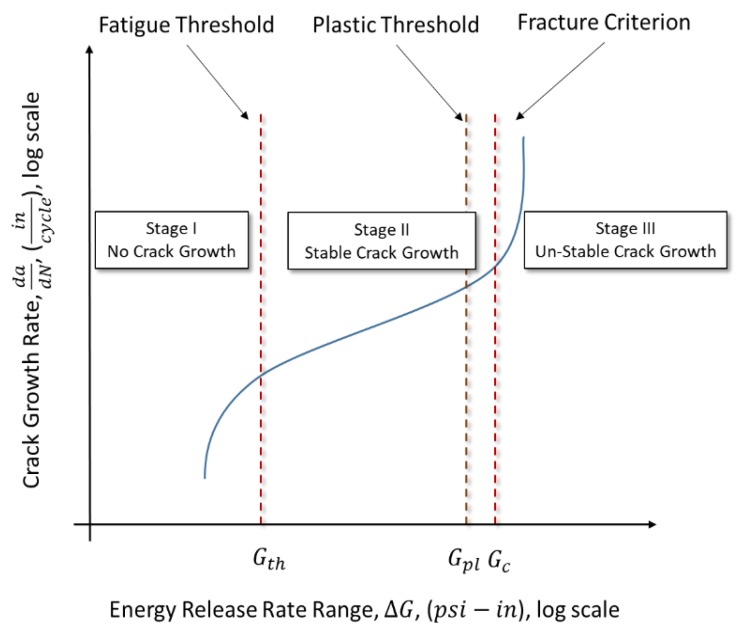
Energy release rate crack growth stages.

Fractures are a separation of a material due to the applied stresses; fatigue and fractures are developed by cyclic stresses over time. These cyclic stresses can be below the yielding point of the material, but as the energy accumulates, it can overcome the capacity of the cohesion energy of the material. The cohesion energy of a material is experimentally determined and commonly expressed as Griffith’s energy release rate threshold (Gth). Manufactured materials are a combination of atoms that have an overall capacity known as the critical energy release rate (Gc). The Gth governs the initial fracture of atomic bonds, while the Gc governs the ultimate exponential crack propagation. In linear elastic fracture mechanics (LEFM), there are three stages of fatigue crack propagation: Stage I, Stage II, and Stage III (Figure 1). Stage I marks the portion before the crack initiates and Stage III is when the crack has reached the Gc of the material and propagates exponentially (Figure 1). In between these two capacities is the section known as Stage II (Figure 1); this is where the crack propagates in a predictable and linear fashion [11,13]. It should also be noted that environmental factors, such as corrosion, play an important role in crack propagation by weakening the material causing faster crack growth [11,13]. 

Over the past several decades, numerous studies have derived numerous methods to describe fatigue and fracture stress and propagation [11,13]. For this article, Griffith’s energy release rate [11], Equation (1), determines the LEFM near-field stresses, Equations (2)–(4), [11], while Paris Law crack propagation [11], Equation (5), calculates the number of cycles, dN, required to propagate a crack over a given distance, da, depending on the material constants C and m.

(1) $$G=\frac{{\sigma^{2}}_{a}\pi a}{E}$$

(2) $$\sigma_{x}=\frac{EG}{\sqrt{2\pi r}}\cos\left(\frac{1}{2}\theta \right)\left(1-\sin\left(\frac{1}{2}\theta \right)\sin\left(\frac{3}{2}\theta \right)\right)$$

(3) $$\sigma_{y}=\frac{EG}{\sqrt{2\pi r}}\cos\left(\frac{1}{2}\theta \right)\left(1+\sin\left(\frac{1}{2}\theta \right)\sin\left(\frac{3}{2}\theta \right)\right)$$

(4) $$\tau_{xy}=\frac{EG}{\sqrt{2\pi r}}\sin\left(\frac{1}{2}\theta \right)\left(\cos\left(\frac{1}{2}\theta \right)\cos\left(\frac{3}{2}\theta \right)\right)$$

(5) $$\frac{da}{dN}=C\Delta G^{m}$$

In Equations (1)–(5), G is the strain energy release rate, $\sigma_{a}$ is the applied far-field stress, a is the crack length (for a center crack, it is equal to the half-crack length), E is the Young’s modulus of the material; $\sigma_{x}$, $\sigma_{y}$, and $\tau_{xy}$ are the near-field stresses at the crack tip; r is the crack tip plasticity radius; and θ is the angle at which $\sigma_{a}$ is applied. 

### 1.2. CFRP Fatigue and Fracture Repairs

**Figure 2:**
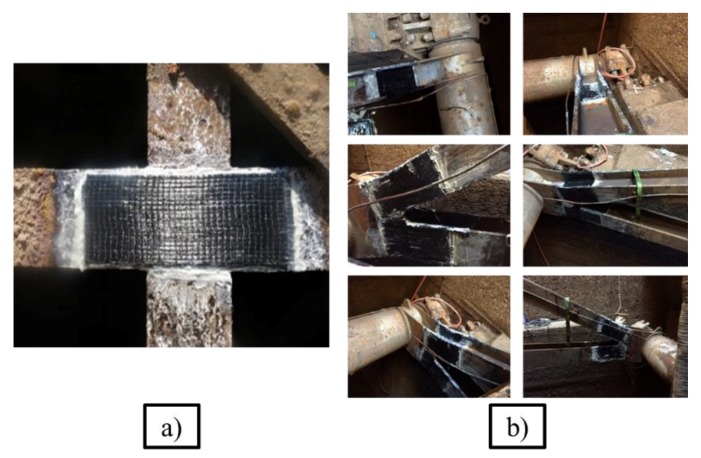
Implemented carbon fiber reinforced polymer (CFRP) repairs. (**a**) Miter gate flange. (**b**) Tainter valve strut arm.

Due to their low density and high strength capacity, CFRP have become a popular method of strengthening and repairing existing structures [9,14]. In recent years, the United States Army Corps of Engineers (USACE) has developed a method to repair NSS with CFRP patches (Figure 2) [8,9]. However, there is still a need to fully understand the chemical bond between steel and CFRP in large steel structures, as most analytical work has been implemented on above ground structures, such as bridges, buildings, and aircrafts [15]. The majority of analytical studies investigate the crack growth behavior of a beam in bending [16,17,18]. Few analytical studies combine the crack growth behavior with the bond-slip behavior of the CFRP patch repairs [19,20,21,22,23]. 

This paper utilizes the experimental results reported in [8] to validate the analytical model. The analytical model is based on the bond contact shear stresses of a single lap joint [12].

### 1.3. Bond

The bonding of two objects allows the transfer of forces through either objects’ surface interface and creates a composite section [24] Adhesives are a common method of joining two surfaces. As long as the applied stresses are below the adhesive’s capacity, the bonded section behaves as a composite. This behavior is beneficial for fatigue crack repair with CFRP patches, over the cracked area, as the overall stress capacity of the system is raised by the addition of the CFRP’s high tensile capacity [25]. This repair methodology introduces an additional strength capacity, reducing the mean stress range at the crack tip [26,27,28].

The majority of studies of the bond behavior of CFRP for crack repair or strengthening a section have been developed for concrete [10,18,23,25]. Some new research has been conducted to describe the behavior of steel and CFRP [19,27,29,30,31,32,33,34]. The majority of the research focuses on only one-directional shear behavior on small scale specimens. 

Several analytical models have been developed to describe the contact shear forces developed between two surfaces (adherends) bonded together by an adhesive as a single lap joint (SLJ). Each analytical model makes certain assumptions that govern the resulting equation. Table 1 gives an overview of the previously mentioned analytical models and their assumptions. In 1938, Volkersen derived a contact shear equation that accounted for the tensile elastic deformation of the adherends and shear-only deformation of the adhesive [12,20]. Volkersen’s model results in a non-uniform shear stress distribution that is greatest at the ends of the bond and lowest in the middle. Most SLJ bonds develop an eccentricity, due to the adhesive’s thickness, developing a bending moment. Several linear elastic extensions to Volkersen’s initial bond behavior analytical model were developed by Goland and Reissner, Frostig et al., Hart-Smith, and Bigwood and Crocombe to model the bending moment and subsequent peeling [20,21].

## 2. Methods

**Figure 3:**
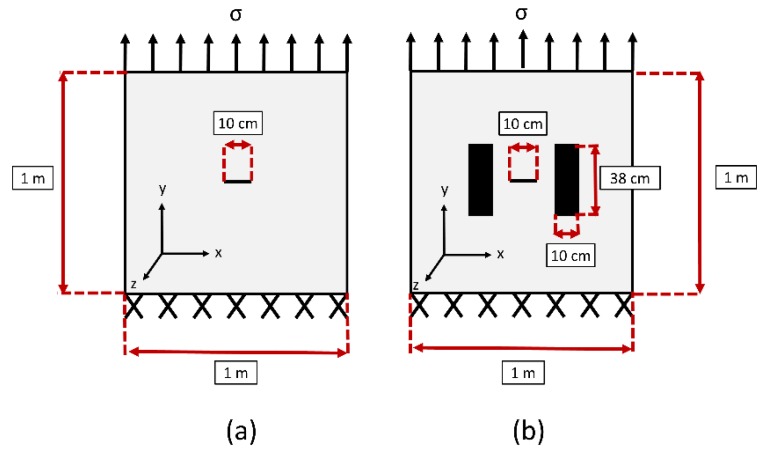
Large-scale steel plate specimen configuration (**a**) with CFRP Repairs; (**b**) without CFRP Repairs.

This paper’s investigation of bond-slip is based on the observations made during testing of a large-scale plate with and without CFRP patch repairs (Figure 3 and Table 2). The article “Underwater Large-Scale Experimental Fatigue Assessment of CFRP-Retrofitted Steel Panels” by Mahmoud et al. gives a full description of the experimental setup and results [8]. During experimentation, Hudco Formula 3200 and Tyfo S epoxy were tested. Hudco Formula 3200 has the ability to cure underwater, while Tyfo S requires dry conditions for curing. The authors in [8] describe how this adhesive is not ideal to increase fatigue life, due to Hudco Formula 3200’s lower strength capacity and faster debonding compared to Tyfo S. The experimental setup was conducted under a Mode I loading of 55 MPa, as reported in [8]. NSSs, such as tainter valves and tainter gates, are subjected to pure Mode I loading.

The CFRP repair method reported in [8] ensured a seamless/no-cold-joint composite of adhesive and CFRP. Therefore, the only bond interface is between the made-up composite and the steel, since the adhesive fully integrates with the CFRP and has a negligible thickness [8]. 

### Analytical

**Figure 4:**
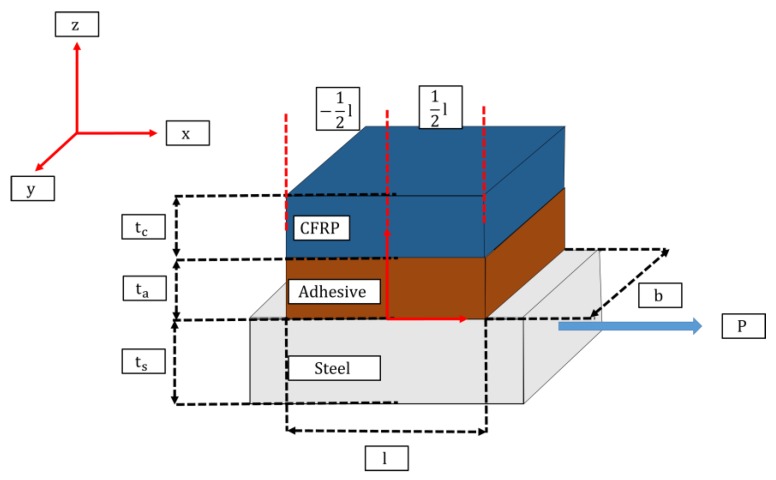
Diagram of single lap joint.

The CFRP was fully saturated when it was adhered to the steel’s surface. This condition eliminated any joints between the CFRP and the adhesive and created a very thin interface between the steel and the CFRP of even stress distribution and no bending moment. The adhesive is also a very brittle adhesive, allowing hardly any deformation. Based on the installation and experimental observations, Volkersen’s shear interface equation was deemed satisfactory. Volkersen’s bond shear derivation is given by Equations (6)–(10).

(6) $$\tau =\frac{P}{bl}\left(\frac{w}{2}\right)\left(\frac{\cosh(wX)}{\sinh\left(\frac{w}{2}\right)}\right)+\frac{\varphi -1}{\varphi +1}\left(\frac{w}{2}\right)\left(\frac{\sinh(wX)}{\cosh\left(\frac{w}{2}\right)}\right)$$

(7) $$w=\sqrt{\left(1+\varphi \right)\vartheta }$$

(8) $$\varphi =\frac{t_{t}}{t_{b}}$$

(9) $$\vartheta =\frac{G_{a}l^{2}}{E_{t}t_{t}t_{a}}$$

(10) $$X=\frac{x}{l},-\frac{1}{2}\leq X\leq \frac{1}{2}$$

where τ is the interface shear; P is the applied load; b is the width of the bond; l is the full overlap; w,φ, and ϑ are defined in Equations (7)–(9), respectively; $t_{t}$, $t_{b}$, and $t_{a}$ are the thicknesses of the top adherend, bottom adherend, and adhesive respectively; $G_{a}$ is the shear modulus of the adhesive, $E_{t}$ is the Young’s modulus of the CFRP adherend; and X is the distance where the shear is being calculated from the center. Figure 4 shows the different dimensions and layers considered for the analytical model. 

This investigation utilized Volkersen’s equation for the contact shear stress, based on the following assumptions. First, the two adherends are highly rigid and experience linear elastic behavior. Second, there is no eccentricity of the adherends. Third, shear and normal stresses of the adhesive are constant through the thickness of the adhesive. Fourth, linear elastic Mode I crack propagation is utilized. Fifth, there is localized debonding of the adhesive at the crack tip as the crack propagates under the CFRP patch. Finally, the fatigue and fracture is described only for linear crack growth.

**Figure 5:**
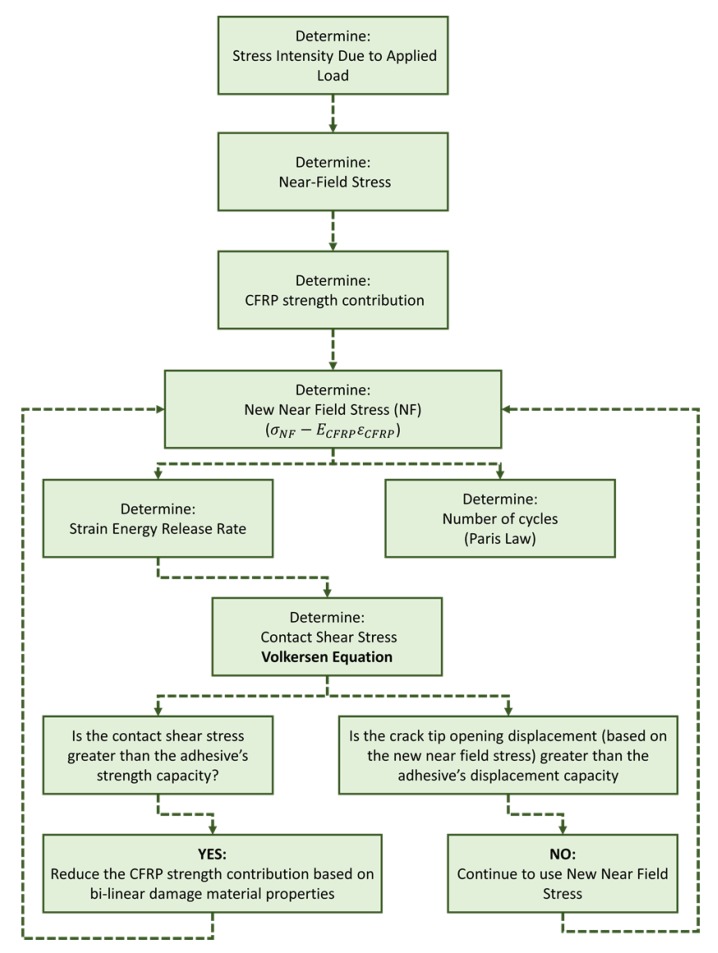
Algorithm of analytical model.

The steps taken to determine the contact shear stresses as the crack propagated are shown in Figure 5. First, the current strain energy release rate, G, was calculated from the externally applied load as the crack grew, using Griffith’s energy theorem (Equation (1)). As a Mode I crack propagates, it develops near-field stresses that can be split into the x and y directions, using Equations (2) and (3) (Figure 5). Then, these x and y stresses, minus the stiffness contribution of the CFRP patch, respectively, result in an x- and y-applied force, used in the Volkersen’s equation (Equation (6)) (Figure 5). 

To determine the contact shear stress, Volkersen’s equation (Equation (6)) utilizes the total surface area of the bond in an SLJ. However, the localized bond stresses of the CFRP/steel interface require a surface area of 0.5% of the total surface area. This reduction in area was applied to Volkersen’s (Equation (6)) in both the x and y directions. If the shear stress in the x and y, respectively, was less than the adhesive’s capacity, then there was no debonding and no bi-linear reduction in the CFRP’s stiffness contribution. However, if the shear stress was greater than the adhesive’s capacity, then the CFRP’s stiffness contribution was reduced. Finally, a new strain energy release rate, G, was calculated with the resultant of the x and y stress to determine the change in cycles as the crack grew, using Paris Law (Equation (5)). 

## 3. Results and Discussion

**Figure 6:**
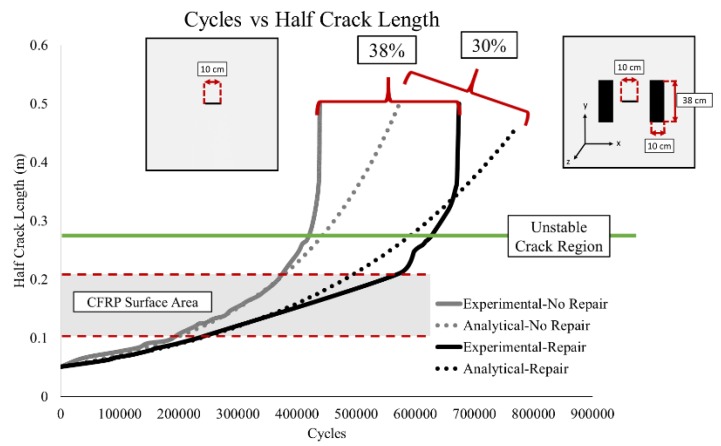
Cycles vs. half crack length results.

Figure 6 shows the cycles versus crack length comparison of the experimental and analytical results for a center through crack large plate with and without CFRP patches. For the unrepaired specimen, the analytical and experimental results match up perfectly, until 350,000 cycles. Then the curves diverge as they approach the unstable crack growth region (Region III of Paris Law) [11]. For the repaired specimen, the results begin to diverge at 375,000 cycles, indicating that during the stable crack growth, the analytical model behaves according to the experimental results. The fatigue life improvement at the beginning of the unstable crack growth rate was 30% and 25% for the experimental and analytical, respectively. Figure 6 also shows a 38% and 30% fatigue life improvement from the experiments and analytical model at fracture, respectively (Figure 6). 

**Figure 7:**
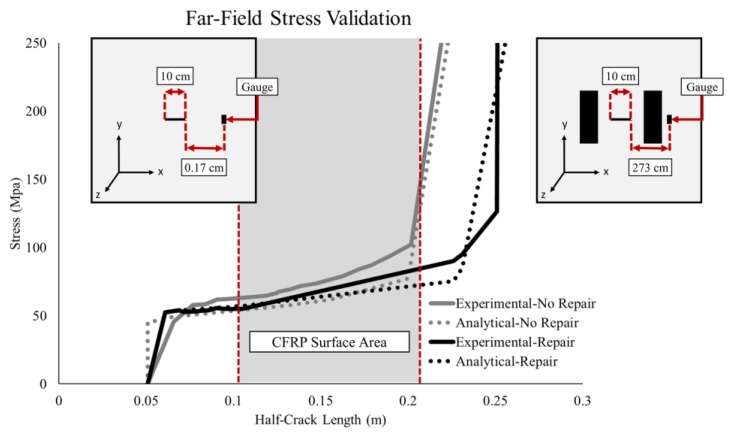
Far-field stress result.

To verify the performance of the analytical model, an additional validation comparing the crack length vs. far-field stresses was performed (Figure 7). For the unrepaired specimen, there is a divergence between the experimental and analytical results as the gauge location is reached. The divergence can be attributed to the sensitivity of the r parameter in the near-field stress equation. However, the stresses converged as the crack approached the strain gauge. The stresses, for the repaired specimen, calculated by the analytical bond model, show a good correlation with the experimental results until the crack growth approaches the strain gauge. The divergence between the repaired experimental and analytical stresses is due to non-linear crack tip plasticity. 

## 4. Conclusions

This paper develops an analytical expansion of Volkersen’s SLJ model [12] to describe the fatigue life improvement of CFRP repairs on cracked steel sections, based on bond-slip behavior. The algorithm required the following assumptions:

- The two adherends are highly rigid and experience linear elastic behavior;
- There is no eccentricity of the adherends;
- Shear and normal stresses of the adhesive are constant through the thickness of the adhesive;
- Linear elastic Mode I crack propagation is utilized;
- The occurrence of the adhesive’s localized debonding at the crack tip as the crack propagates under the CFRP patch;
- Fatigue and fracture linear crack growth.

The assumptions allowed the determination of localized debonding, based on the applied far-field stresses. 

The analytical model was validated against the experimental number of cycles vs. crack length and far-field stresses vs. crack length data. The cycles vs. crack length results demonstrated a fatigue life improvement of 25% at the start of the unstable region and 30% at fracture. Experimental far-field stresses supplied further validation. It was noted that the unrepaired analytical results diverged from the experimental results due to the sensitivity in the r variable of the near-field stresses at the crack tip. The repaired analytical model’s stress results demonstrated a good correlation with the experimental results until the crack began to approach the strain gauge location. The divergence between the repaired experimental and analytical stresses is due to non-linear crack tip plasticity. The analytical model was determined to capture the extended fatigue life behavior of CFRP repairs of steel cracking, which have been proven to improve the fatigue life in NSS by [8,9]. 

## Figures and Tables

**Table 1:** Summary of common bond shear analytical models and their common assumptions [21].

Analytical Model	Assumptions				
	Linear Elastic	Deformation	Bending	Transverse Stress	Type of Joint ^1^
Volkersen	x	Shear only		Equal	SLJ & DLJ
Goland and Reissner	x	Shear & Normal	x	Equal	SLJ & DLJ
Frostig et al.	x	Shear & Normal	x	Equal	SLJ & DLJ
Hart-Smith	x	Shear & Normal	x	Equal	SLJ & DLJ
Bigwood and Crocombe	x	Shear & Normal	x	Equal	Multiple Configuration

^1^ Types of joints: Single Lap Joint (SLJ) and Double Lap Joint (DLJ).

**Table 2:** Material properties.

Mechanical Property	Steel	Tyfo SCH-41 (Carbon Fiber) [35]	Tyfo S (Adhesive) [36]
Ex ^1^ (Gpa)	-	5.93	-
Ey ^1^ (Gpa)	206	82.7	2.9
Gxy (Gpa)	-	29.5	1.03
ε	-	0.0085	-
σy (Gpa)	344.7	82	-
τu (Gpa)	-	-	1.69 × 10^−2^
Gc (Gpa) (Fracture Energy)	-	-	2.10 × 10^−7^

^1^ The x and y denote the latitudinal and longitudinal directions, respectively.
